# Gremlin-1 augments the oestrogen-related receptor α signalling through EGFR activation: implications for the progression of breast cancer

**DOI:** 10.1038/s41416-020-0945-0

**Published:** 2020-06-23

**Authors:** Sin-Aye Park, Nam Ji Sung, Bae-Jung Choi, Wonki Kim, Seung Hyeon Kim, Young-Joon Surh

**Affiliations:** 1 Department of Biomedical Laboratory Science, College of Medical Sciences, Soonchunhyang University, Asan, 31538 South Korea; 2 Tumor Microenvironment Global Core Research Center, College of Pharmacy, Seoul National University, Seoul, 08826 South Korea; 3 Department of Molecular Medicine and Biopharmaceutical Sciences, Graduate School of Convergence Science and Technology, Seoul National University, Seoul, 08826 South Korea; 4 Cancer Research Institute, Seoul National University, Seoul, 03080 South Korea

**Keywords:** Breast cancer, Breast cancer

## Abstract

**Background:**

Gremlin-1 (GREM1), one of the bone morphogenetic protein antagonists, is involved in organogenesis, tissue differentiation and kidney development. However, the role of GREM1 in cancer progression and its underlying mechanisms remain poorly understood.

**Methods:**

The role of GREM1 in breast cancer progression was assessed by measuring cell viability, colony formation, 3D tumour spheroid formation/invasion and xenograft tumour formation. Chromatin immunoprecipitation, a luciferase reporter assay and flow cytometry were performed to investigate the molecular events in which GREM1 is involved.

**Results:**

GREM1 expression was elevated in breast cancer cells and tissues obtained from breast cancer patients. Its overexpression was associated with poor prognosis in breast cancer patients, especially those with oestrogen receptor (ER)-negative tumours. *GREM1* knockdown inhibited the proliferation of breast cancer cells and xenograft mammary tumour growth, while its overexpression enhanced their viability, growth and invasiveness. Oestrogen-related receptor α (ERRα), an orphan nuclear hormone receptor, directly interacted with the *GREM1* promoter and increased the expression of GREM1. GREM1 also enhanced the promoter activity of *ESRRA* encoding ERRα, comprising a positive feedback loop. Notably, GREM1 bound to and activated EGFR, a well-known upstream regulator of ERRα.

**Conclusions:**

Our study suggests that the GREM1–ERRα axis can serve as a potential therapeutic target in the management of cancer, especially ER-negative tumour.

## Background

Breast cancer is one of the most frequent causes of cancer-related death among women worldwide. Although the current treatments for breast cancer have been substantially improved, the majority of patients still experience severe side effects, therapeutic resistance and tumour metastasis.^1,2^ Determination of the oestrogen receptor (ER) status of breast carcinomas is essential for defining therapeutic procedures, and ER is considered as a successful target for the treatment of ER-positive breast cancer. In contrast, ER-negative breast tumours do not have definitive molecular targets and show poor prognosis compared to ER-positive tumours.^3,4^ Thus, more effective targeted therapeutics need to be developed in the management of ER-negative breast cancer.

Gremlin-1 (GREM1) is a member of the cystine knot superfamily and a bone morphogenetic protein (BMP) antagonist.^5,6^ GREM1 plays a critical role in embryogenesis, organ development and tissue differentiation through regulation of BMPs.^7,8^ In addition, GREM1 has been involved in diverse pathological conditions, such as renal^9,10^ or pulmonary fibrosis,^11,12^ renal inflammation^13,14^ and diabetic kidney disease^15,16^ in BMP-dependent or -independent manners. Above all, GREM1 is well known to induce fibrosis of organs, which requires the epithelial–mesenchymal transition (EMT) process.^9,17^

Since EMT is an important step in cancer metastasis,^18^ its induction by GREM1 may affect tumour progression. GREM1 is overexpressed in human tumours, including carcinomas of the colon, lung, ovary, sarcoma, and pancreas.^19–21^ According to multiple microarray-based studies, the level of the GREM1 gene expression was found to be highly elevated in breast tumours and tumour stroma.^4,22–25^ However, the functions of GREM1 in breast cancer progression and underlying mechanisms remain largely unresolved.

Oestrogen-related receptor α (ERRα) is an orphan nuclear hormone receptor with no known endogenous ligand. Despite a high degree of similarity between ERRα and the ERα, the activity of these two receptors is regulated by distinct molecular mechanisms.^26^ ERRα interacts with nuclear receptor co-activators, such as proliferator-activated receptor γ coactivator 1 (PGC-1), without binding to a natural oestrogen.^27,28^ The results of chromatin immunoprecipitation (ChIP) combined with microarray analysis showed that most of the genes regulated by ERRα were distinct from those regulated by ERα.^29^

ERRα has been identified as a potent prognostic factor^30^ and a therapeutic target^31^ in human breast cancer. ERRα has been reported to modulate breast cancer cell metabolism, growth, and proliferation through regulation of multiple oncoproteins.^26,32^ ERRα is especially critical for the growth of ER-negative^33^ or triple (ER, progesterone receptor, and HER2/neu)-negative breast cancer cells.^34,35^ The transcriptional activity of ERRα in breast cancer is known to be regulated by receptor tyrosine kinases, such as ERBB2, commonly referred to as HER2,^36^ and also by epidermal growth factor receptor (EGFR). ^26^

Herein, we report the oncogenic role of GREM1 in breast cancer growth and progression. Notably, GREM1 induces transcriptional activity of ERRα through EGFR activation, which in turn upregulates GREM1 expression.

## Methods

### Cells and reagents

MCF-10A, MCF-10A-*ras*, MDA-MB-453, MDA-MB-468, SKBR3, MCF-7, T47D, and CCD-1068sk cells were originally obtained from American Type Culture Collection, and the BT474 cell line was obtained from Korean Cell Line Bank. The cells were cultured according to the standard procedure and maintained at 37 °C in a humidified atmosphere composed of 5% CO_2_/95% air. GREM1 antibody was purchased from Abcam and recombinant human GREM1 was obtained from R&D systems. Recombinant human EGF, anti-Flag antibody and cell linker kits (PKH26 and PKH67) were purchased from Sigma-Aldrich. Anti-ERRα, anti-p-EGFR/EGFR, anti-p-Akt/Akt, anti-p-ERK/ERK antibodies and erlotinib were obtained from Cell Signalling Technology. Expression plasmids of GREM1, ERRα, Flag-only, Flag-EGFR, and Flag-BMP2 were purchased from Sino Biological Inc. Fc-IgG1 and Fc-GREM1 were provided by ACROBiosystems. The lentiviral GREM1 clone was obtained from Genecopoeia. 3xERRE-luciferase, pcDNA4-myc-PGC-1α, EGFR-WT, EGFR-ECD and EGFR-ICD were provided by Addgene. XCT790, LY294002 and U0126 were purchased from Tocris.

### Gene silencing

Endogenous ERRα was knocked down using specific small interfering RNAs (siRNAs) (Life Technologies, Assay ID# 289481 and 5089). Briefly, cells were transiently transfected with siRNAs by reverse transfection using Lipofectamine RNAiMAX (Life Technologies). Two TRC lentiviral GREM1 short hairpin RNAs (shRNAs) (TRCN0000063834 and TRCN0000063837) and shControl (shCtrl; TRC ID# SHC002) were obtained from Dharmacon, and the lentiviruses were packaged in 293 T cells. The cells were transiently transfected with shRNA vector together with pCMV-VSV-G and pCMV-dR8.91 using Lipofectamine 2000 (Life Technologies). After transfection for 72 h, the viral supernatant was collected, filtered and used for the transduction of breast cancer cells in the presence of 8 μg/ml polybrene (Merck Millipore). Stable cells were selected by 1 μg/ml puromycin (InvivoGen).

### Cell proliferation assay

Cells were plated in 96-well plates (2 × 10^3^/well) and incubated for 3 days. The cells were then treated with MTT (0.5 mg/ml) for 4 h at 37 °C, and 100% dimethyl sulfoxide was added to dissolve the crystals. Viable cells were counted by reading the absorbance at 570 nm using a microplate reader SpectraMax (Molecular Devices). For the colony formation assay, 2 × 10^3^ cells were plated in the 6-well plates and allowed to grow for 7 to 10 days. After the medium was removed, cells were fixed with 10% formalin for 15 min, and stained with crystal violet to visualise the colonies.

### Quantitative real-time PCR

Total RNA was isolated from cells using TRIzol® (Invitrogen). Reverse transcription of total RNA was performed using the M-MLV reverse transcriptase (Promega). Quantitative PCR (qPCR) was performed using qPCR reagents (Nanohelix) and 7500 Real-Time PCR (Applied Biosystems). Primer sequences are listed in the Supplementary Table S1.

### Western blot analysis

Standard sodium dodecyl sulfate-polyacrylamide gel electrophoresis (SDS-PAGE) and Western blotting procedures were used to analyse the expression of various proteins. Cell lysates were prepared using SDS lysis buffer (50 mM Tris-HCl, pH 6.8, 2% SDS, 10% glycerol, and 0.02% bromophenol blue) containing protease inhibitors and phosphatase. All proteins were visualised using a horseradish peroxidase-conjugated secondary antibody (GE Healthcare Life Sciences) and AbSignal detection reagents (AbClone).

### 3D tumour spheroid formation assay

First, breast fibroblast CCD-1068sk cells and breast cancer SKBR3 cells were labelled with a green and a red fluorescent cell linker (Sigma-Aldrich), respectively. Labelled cells (2 × 10^3^ cells/well) were combined, seeded, and incubated in 3D hanging-drop 96-well plates for 5 days. The spheroids were visualised under the fluorescence microscope, and their sizes were quantified.

### 3D tumour spheroid invasion assay

The cells containing spheroid formation extracellular matrix were seeded in the 3D culture qualified 96-well spheroid formation plate (CULTREX). After incubation for 3 days, the invasion matrix and medium containing 20% foetal bovine serum (FBS) alone, 20% FBS with EGF (50 ng/ml) or 20% FBS with GREM1 (50 ng/ml) were added. The mixtures were incubated for additional 7 days, and the invasion of spheroids was observed under the microscope. The obtained images were analysed using the ImageJ software as described by the manufacturer.

### ChIP assay

The SimpleChIP Enzymatic kit (Cell Signalling) was used as described by the manufacturer. PCR was performed with primers specific for the indicated promoter regions. The reactions were run in triplicate, and 1% of the total input sample was used as a control. Primer sequences are listed in the Supplementary Table S2.

### Luciferase reporter gene assay

Cells were plated in 24-well plates for 24 h and co-transfected with expression plasmids of ERRα and PGC-1α as well as 3xERRE-luciferase and Renilla-luciferase construct using Lipofectamine 2000 for additional 48 h. Luciferase reporter gene assays were performed using the Dual-Luciferase Reporter Assay System (Promega) according to the manufacturer’s instructions.

### Immunofluorescence staining

For staining fixed paraffin-embedded tissues, a standard protocol for deparaffinization, antigen retrieval, and permeabilisation was followed. The tissues were incubated overnight with anti-GREM1 antibody at 4 °C, washed with phosphate-buffered saline (PBS), and incubated further with Alexa Fluor 488 antibody (Invitrogen) for 1 h at room temperature. After washing, the tissues were stained using ProLong® Gold Antifade Reagent containing DAPI (4′,6-diamidino-2-phenylindole; Invitrogen).

### Flow cytometry

HEK293 cells were transiently transfected with Flag alone, Flag-EGFR or Flag-BMP2 for 48 h, followed by incubation with Fc-IgG1 (Fc-control) or Fc-GREM1 (3 μg/ml, each) for 1 h at 4 °C. After washing with PBS, the cells were incubated with 2 μg/ml of Alexa Fluor 647 goat anti-human IgG antibody (Life Technologies) for 30 min at 4 °C. After washing with PBS, the cells were passed through cell strainer to eliminate clumps. Flow cytometry was performed using a FACS Canto II instrument (BD Biosciences).

### Immunoprecipitation assay

HEK293 cells were transiently transfected with Flag alone, Flag-EGFR, or Flag-BMP2 for 48 h, followed by incubation with Fc-IgG1 (Fc-control) or Fc-GREM1 (100 ng/ml, each) for additional 1 h at 37 °C. Cell lysates were prepared with IP Lysis Buffer (Thermo Scientific) and subjected to immunoprecipitation using anti-Flag antibody with conjugated protein A/G PLUS-agarose (Santa Cruz Biotechnology). Immune complexes were subjected to Western blot analysis and detected with anti-Fc antibody (Jackson ImmunoResearch Laboratories).

### Animal study

Female BALB/c (nu/nu) athymic nude mice, 5 weeks of age (weight 17–19 ± 1–2 g), were purchased from Orient Bio Inc. Mice were maintained in specific pathogen-free conditions: 20–24 °C, 12/12 h of dark/light cycle, 60 ± 5% of humidity, and plastic cage (3–4 mice/cage). Bedding materials were changed every week, and environmental enrichment was done with sterile materials. All animal experiments were approved by the Institutional Animal Care and Use Committee of the Seoul National University Ethics Research Board (Permit number: SNU-170511-1-1). To minimise the number of mice and the effects of subjective bias when injecting cells into mice and evaluating the results, two different cell lines (7 × 10^6^ cells/flank) were inoculated subcutaneously into the right and left dorsal flanks of female nude mice after isoflurane inhalation anaesthesia. Animals were assigned to groups after randomisation. Experimental groups are as follows: six mice of SKBR3-shCtrl (left flank) and SKBR3-shGREM1 (right flank); four mice of SKBR3-mock (left flank) and SKBR3-GREM1 (right flank). Tumour volume (0.52 × length × width^2^) and body weights were measured three times a week. At the end of the experiments, mice were euthanized by CO_2_ inhalation and each tumour was removed.

### Statistical analysis

Data were expressed as the mean ± SD of the results obtained from at least three independent experiments. Independent-sample two-sided Student’s *t* test was used to compare two groups with normal distribution data and a *P* value of <0.05 was considered to be statistically significant. *P < 0.05, **P < 0.01, and ***P < 0.001.

## Results

### GREM1 is overexpressed in human breast cancer, which is associated with worse survival of breast cancer patients

**Fig. 1:**
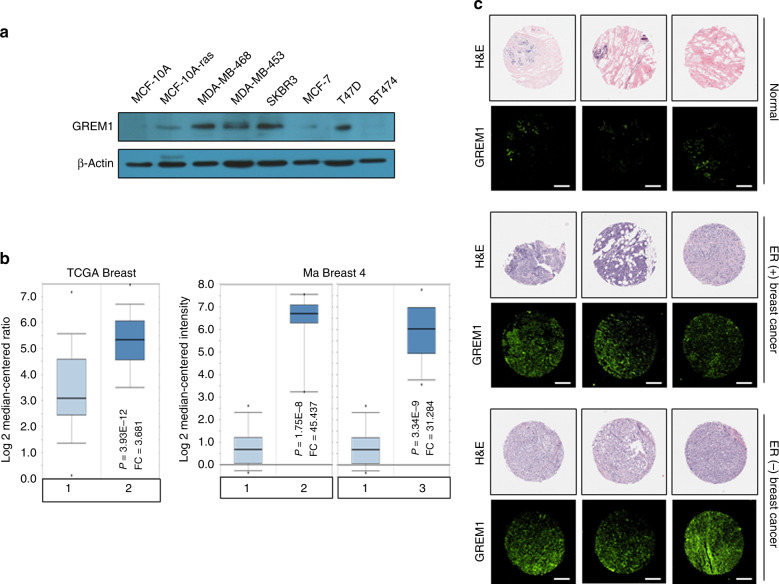
GREM1 expression is upregulated in human breast cancer. **a** Expression levels of GREM1 in human breast cancer cells. MCF-10A cells were used as a normal control. The protein expression was determined by immunoblot analysis. **b** Oncomine plots of *GREM1* mRNA levels in two sets of data, TCGA Beast and Ma Breast 4. TCGA Breast [Group 1, normal breast (*n* = 61); Group 2, invasive breast carcinoma (*n* = 76)]. Ma Breast 4 [Group 1, normal breast (*n* = 14); Group 2, invasive ductal breast carcinoma (n = 9); Group 3, ductal breast carcinoma in situ (*n* = 11)]. *P*, *P* value; FC, fold change. **c** Representative immunostains for GREM1 expression in normal, ER-positive breast and ER-negative breast cancer tissues. The human breast cancer tissue microarray and haematoxylin and eosin (H&E) images were provided by US Biomax Inc. (Cat# BR1009). Scale bar = 200 μm.

As an initial approach to assess a role of GREM1 in breast cancer development, we first examined the level of GREM1 in some transformed or cancerous human breast cell lines as compared to non-cancerous human breast epithelial cells (MCF-10A). The protein expression of GREM1 was found to be markedly elevated in breast cancer cell lines, particularly ER-negative ones, including MDA-MB-468, MDA-MB-453 and SKRB3 cells (Fig. 1a). We next utilised a cell line database (Expression Atlas, https://www.ebi.ac.uk/gxa/home) to compare the expression of *GREM1* among multiple breast cancer cell lines. Among the 69 breast cancer cell lines, the ER-negative cell lines were 48 and ER-positive cell lines were 21. In this database, the level of *GREM1* expression in each cell line was analysed by Transcripts Per Million unit, and the proportion of cell lines in which *GREM1* expression was significantly upregulated was 45.83% (22/48) for ER-negative cell lines, but 0% (0/21) for ER-positive cell lines (Supplementary Excel File 1). Extracellular GREM1 was detected in the collected conditioned media from control breast cancer cells (MDA-MB-453-shCtrl and SKBR3-shCtrl), while it was undetectable in GREM1-depleted cells (MDA-MB-453-shGREM1 and SKBR3-shGREM1). In addition, the level of extracellular GREM1 secretion was increased in non-cancerous human mammary epithelial MCF-10A cells overexpressing GREM1 (MCF-10A-GREM1) compared to control MCF-10A cells (Supplementary Fig. S1).

**Fig. 2:**
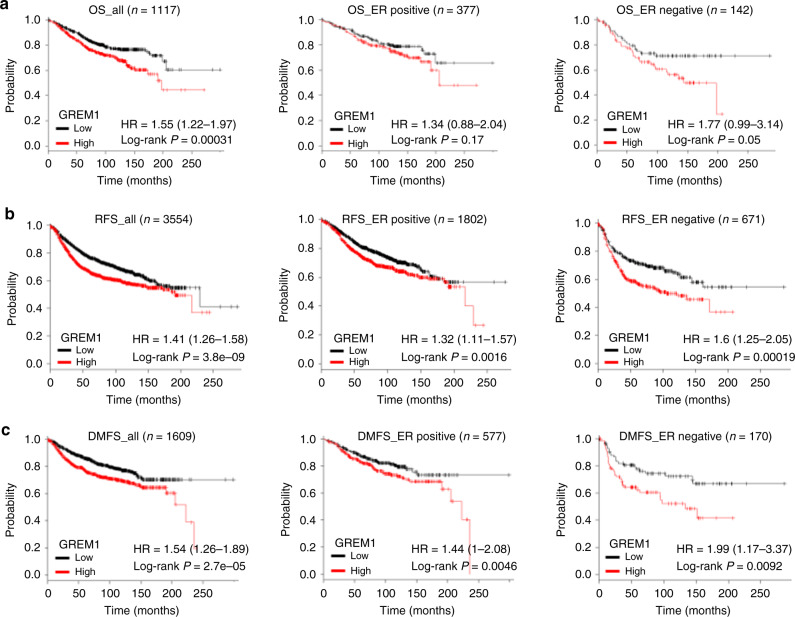
High *GREM1* expression is associated with worse outcome in human breast cancer. **a**–**c** Kaplan–Meier analysis (http://kmplot.com/analysis) of OS (**a**), RFS (**b**) or DMFS (**c**) by low or high *GREM1* mRNA (GREM1 probe set 218468_s_at) expression in each indicated number of breast cancer patients. OS, overall survival; RFS, relapse-free survival; DMF, distant metastasis-free survival; HR, hazard ratio.

To determine the clinical relevance of GREM1 expression to breast cancer progression, we used the Oncomine database. *GREM1* expression was significantly elevated in invasive or ductal breast carcinoma in situ compared to normal tissues in two data sets, TCGA Breast and Ma Breast 4^22^ (Fig. 1b). However, the Oncomine results showed no significant difference in *GREM1* expression between ER-negative and ER-positive patients (Supplementary Fig. S2). Immunofluorescence staining of human breast cancer tissue microarrays showed that *GREM1* was overexpressed in ER-positive and ER-negative breast cancer tissues compared to normal breast tissues (Fig. 1c). Fluorescence intensity for each stain was measured and analysed quantitatively (Supplementary Table S3). Of note, the overexpression of *GREM1* was associated with reduced overall survival (OS), especially in ER-negative breast cancer patients (*GREM1* low vs. high expression patients: hazard ratio (HR) of survival = 1.77, 95% confidence interval (CI): 0.99–3.14, *P* = 0.05) (Fig. 2a). The *GREM1* messenger RNA (mRNA) level was also closely associated with worse relapse-free survival (RFS, *GREM1* low vs. high expression patients: HR of survival = 1.6, 95% CI: 1.25–2.05, *P* = 0.00019) (Fig. 2b) and distant metastasis-free survival (DMFS, *GREM1* low vs. high expression patients: HR of survival = 1.99, 95% CI: 1.17–3.37, *P* = 0.0092) (Fig. 2c) in ER-negative breast cancer patients. However, the level of *GREM2*, a paralog of *GREM1*, was not associated with OS, RFS and DMFS in breast cancer patients (Supplementary Fig. S3). These data suggest that abnormal upregulation of GREM1 is closely related to the mortality of patients with ER-negative breast cancer.

### GREM1 contributes to the oncogenicity of breast cancer cells

**Fig. 3:**
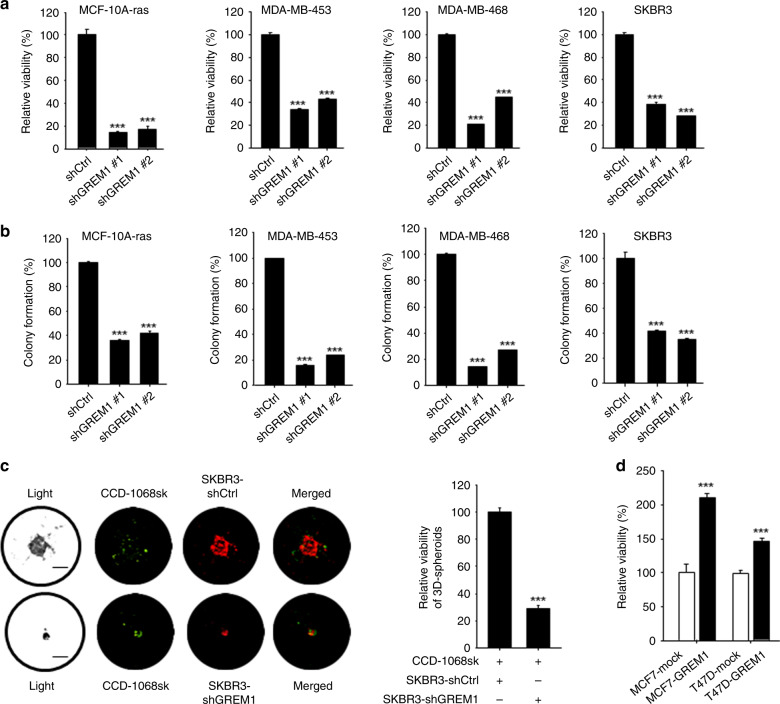

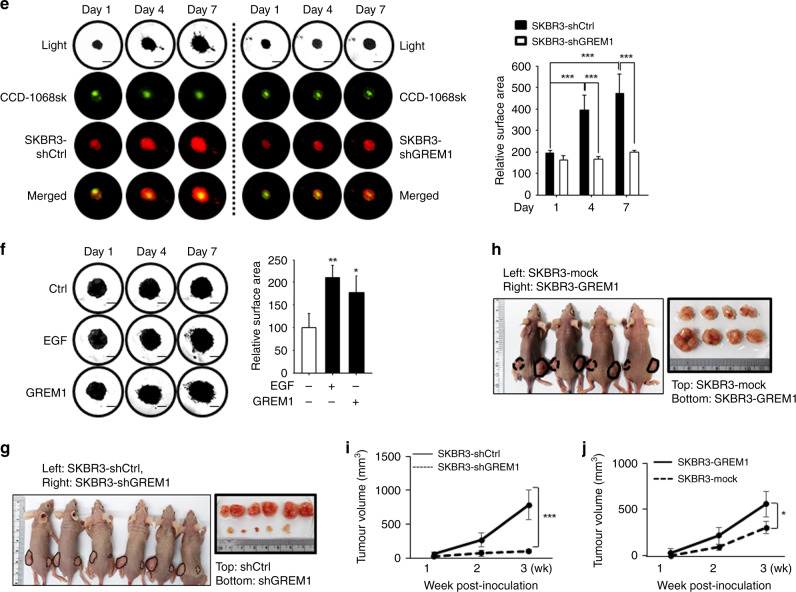
GREM1 regulates the growth of breast cancer cells in vitro and in vivo. **a** Relative viability of transformed and cancerous breast cells. Cells were seeded in 96-well plates and incubated for 72 h, followed by the MTT assay. **b** Colony-forming activity of transformed and cancerous breast cells. Cells were seeded in 6-wells with low density (2 × 10^3^/well) and incubated for 7 to 10 days. **c** Effects of *GREM1* silencing on spheroid formation. CCD-1068sk breast fibroblasts and SKBR3 human breast cancer cells were stained with green and red fluorescent cell linker dyes, respectively, and seeded in 96-well hanging-drop plates for 5 days. The size of each spheroid was measured by fluorescence microscopy, and the cell viability was examined by the MTT assay. **d** Effects of ectopic overexpression of GREM1 on viability of MCF-7 and T47D cells. Corresponding cells were seeded in 96-well plates, incubated for 72 h, and subjected to the MTT assay. **e** Suppressive effects of *GREM1* silencing in a 3D spheroid invasion assay. CCD-1068sk and SKBR3 cells were stained with fluorescent dyes as described in  **c**, suspended in the spheroid formation extracellular matrix and seeded in spheroid formation plate. After 3-day incubation, cell culture medium containing invasion matrix was added and further incubated at 37 °C for additional 7 days. The spheroids were visualised under a fluorescence microscope and each image was analysed using ImageJ to measure changes in the area of the invasive structures. **f** Effect of exogenous GREM1 on the 3D spheroid invasion of SKBR3 cells. SKBR3 cells were suspended in the spheroid formation extracellular matrix and incubated for 3 days. The invasion matrix and cell culture medium containing recombinant human EGF or GREM1 (50 ng/ml, each) were then added, and the mixtures were incubated at 37 °C for additional 7 days. The images were analysed using ImageJ. **g**, **h** Representative images of dissected xenogeneic tumours in nude mice inoculated with *GREM1*-silenced (**g**) or overexpressing (**h**) SKBR3 breast cancer cells. These cells were inoculated subcutaneously into the right dorsal flanks of female nude mice while their respective control cells were inoculated into the left dorsal flanks as described in ‘Methods’ section. Representative images of dissected xenogeneic tumours from nude mice in each group. **i**, **j** Quantification of tumours. Female nude mice were treated as described for **g**, **h**. All values in the graphs represent mean ± SD of three independent experiments except for **i**, **j**, where *n* = 6 and 4 per group, respectively. Two-sided *t* test. *P < 0.05; **P < 0.01; ***P < 0.001.

Next, we investigated the role of GREM1 in the growth of breast cancer cells. For this purpose, we utilised the lentiviral shRNA system to establish stable cell lines in which GREM1 expression was inhibited. *GREM1* knockdown significantly suppressed the viability (Fig. 3a) and the colony formation (Fig. 3b) of multiple ER-negative human breast cancer cell lines as well as H-*ras*-transformed human mammary epithelial cells. In addition, we performed a GREM1 rescue experiment with the MDA-MB-453 breast cancer cell line to ensure that the effect of shGREM1 was not due to the off-target effect. As shown in Supplementary Fig. S4, the 72-h viability of *GREM1*-knockdown MDA-MB-453-shGREM1 cells was 53.2%, compared to that of the control cells (shCtrl). When *GREM1* was overexpressed in shGREM1 cells, the survival rate was significantly restored (Supplementary Fig. S4). However, *GREM1* knockdown had little effect on the viability of non-cancerous breast epithelial MCF-10A cells (Supplementary Fig. S5a, b). The 3D tumour spheroid analysis showed that the sizes of the spheroids formed by co-culturing CCD-1068sk breast fibroblasts and SKBR3-shGREM1 breast cancer cells were much smaller than those of the spheroids formed by co-culturing CCD-1068sk and SKBR3-shCtrl cells (Fig. 3c). Ectopic expression of *GREM1* in ER-positive MCF-7 and T47D cells resulted in increased viability as compared to mock vector-transfected control cells (Fig. 3d). In contrast, silencing of *GREM1* expression in the ER-positive T47D cell line also decreased the cell viability (Supplementary Fig. S5c, d), but not as much as that achieved in ER-negative cell lines.

The invasive capability of the spheroids derived from *GREM1*-silenced SKBR3 breast cancer cells co-cultured with CCD-1068sk breast fibroblasts was significantly reduced compared to that of the spheroids formed by co-culture of CCD-1068sk and control breast cancer (SKBR3-shCtrl) cells (Fig. 3e). Conversely, direct treatment with human recombinant GREM1 protein enhanced the invasiveness of SKBR3 cells to the extent equivalent to that achieved with EGF, which was included as a positive control (Fig. 3f). The protein expression levels of GREM1 in all cell lines used in the entire experiments are shown in Supplementary Fig. S6.

After the role of GREM1 in proliferation and invasiveness of transformed or cancerous human breast epithelial cell lines was confirmed, the effect of GREM1 on tumour formation in vivo was investigated in a xenograft mouse model. BALB/c athymic nude mice received the subcutaneous injection of control, *GREM1*-silenced or *GREM1*-overexpressing SKBR3 breast cancer cells. Tumours were excised 3 weeks after breast cancer cell inoculation, and their images were photographed (Fig. 3g, h). *GREM1*-knockdown cells showed much lower tumour growth rates than control cells with dramatic reduction in the tumour volume [mean ± SD (mm^3^): 790.412 ± 219.830 (SKBR3-shCtrl) vs. 97.782 ± 27.008 (SKBR3-shGREM1), 6 xenografts/each cell line] (Fig. 3i), whereas overexpression of *GREM1* resulted in significantly increased tumour volume [mean ± SD (mm^3^): 304.496 ± 65.917 (SKBR3-mock) vs. 559.187 ± 137.707 (SKBR3-GREM1), 4 xenografts/each cell line)] (Fig. 3j). During the experiment, the body weights of the mice did not differ significantly between groups, and no subjects significantly lost their weight. Taken together, these findings clearly demonstrate that GREM1 is essential for the growth and progression of breast cancer cells.

### ERRα binds to the *GREM1* promoter and regulates GREM1 expression

**Fig. 4:**
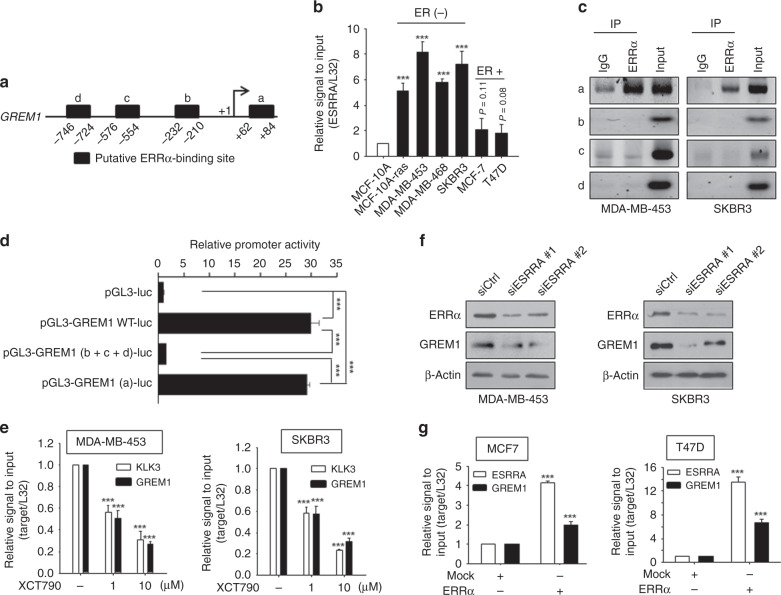
ERRα increases GREM1 expression. **a** Schematic diagram showing the positions of putative ERRα binding elements located in the promoter of human *GREM1* (http://www.genomatix.de). **b** Relative mRNA levels of *ESRRA* in human breast cancer cells. The expression level was quantitated by qPCR analysis. **c** ChIP assay. The binding of ERRα to the *GREM1* promoter was detected by visualisation of the PCR product. Each PCR primer set was designed for the putative ERRα binding elements ‘a’ to ‘d’. **d** Relative *GREM1* promoter activity measured with different GREM1-luciferase (luc) constructs. HEK293 cells were transfected with pGL3-GREM1-luc, which includes all elements, pGL3-GREM1(b + c + d)-luc, which lacks the region ‘a’, or pGL3-GREM1(a)-luc. Luciferase activity was measured and normalised by Renilla activity. pGL3-luciferase construct (pGL3-luc) was used as a negative control. **e** Effect of XCT790 on *GREM1* expression. Cells were treated with XCT790 (1 or 10 μM) for 24 h, and the mRNA levels of KLK3 and GREM1 were quantitated by qPCR analysis. **f** Effect of *ESRRA* knockdown on GREM1 expression. Cells were transfected with siCtrl or two siESRRAs (40 nM, each) for 48 h and the lysates were immunoblotted with the indicated antibodies. **g** Effect of ERRα overexpression on *GREM1* expression. Cells were transfected with mock or ERRα in the presence of PGC-1α for 48 h and the mRNA levels of genes were quantified by qPCR analysis. All values in the graphs represent mean ± SD of three independent experiments. Two-sided *t* test. ***P < 0.001.

In a subsequent study, we examined the promoter sequences of *GREM1* to identify a transcription factor responsible for regulating its expression. For this purpose, we sorted the transcription factors that are likely to bind to the promoter of the human *GREM1* gene by searching for the target gene databases (Supplementary Excel File 2). Of the candidate transcription factors identified, we focused on ERRα (gene name, ‘*ESRRA*’) because of its crucial role in growth of ER-negative breast cancer.^33^ We were able to identify several putative ERRα binding motifs on the promoter of *GREM1* gene within the 1 kb region from the transcription start site (+1) (Fig. 4a). Similar to *GREM1*, the level of *ESRRA* was higher in ER-negative breast cancer cells than in ER-positive ones as well as non-oncogenic MCF-10A cells (Fig. 4b). To determine whether ERRα could directly bind to the human *GREM1* promoter, the ChIP assay was performed by immunoprecipitation with ERRα antibody and IgG as a negative control. The sequences of ERRα putative binding sites used in the ChIP analysis are provided in Supplementary Table S4. As illustrated in Fig. 4c, only the primer set that covers the binding motif ‘a’ (+62 to +84) exhibited strong ERRα interaction with the *GREM1* promoter in both MDA-MB-453 and SKBR3 cells, but there was no ERRα binding observed in the other sites (b–d). More importantly, deletion of the ‘a’ motif [pGL3-GREM1 (b + c + d)-luc] abolished the *GREM1* promoter activity, whereas the construct [pGL3-GREM1 (a)-luc] harbouring only the ‘a’ element had the activity similar to that of the pGL3-GREM1 WT-luc (Fig. 4d). These results suggest that ERRα preferentially binds to this specific site, thereby regulating the expression of GREM1.

XCT790 is a potent and specific antagonist of ERRα capable of inhibiting the constitutive activity of ERRα. Treatment with XCT790 significantly reduced the mRNA level of GREM1 as well as that of KLK3, one of the ERRα target genes, in MDA-MB-453 and SKBR3 breast cancer cells (Fig. 4e). The effective concentration (10 μM) of XCT790 significantly reduced both expression of GREM1 and viability of these breast cancer cells (Supplementary Fig. S7a). In line with above findings, the mRNA (SupplementaryFig. S7b) and protein (Fig. 4f) expression levels of GREM1 were also reduced by siRNA silencing of *ESRRA* encoding ERRα.

Conversely, overexpression of ERRα greatly enhanced the mRNA level of GREM1 in MCF-7 and T47D cells (Fig. 4g). ERRα overexpression also increased the protein expression of GREM1 in SKBR3 cells (Supplementary Fig. S7c). Altogether, these data suggest that ERRα is a potent transcriptional regulator of GREM1 and that *GREM1* is a novel target gene of ERRα.

### GREM1 activates EGFR signalling

**Fig. 5:**
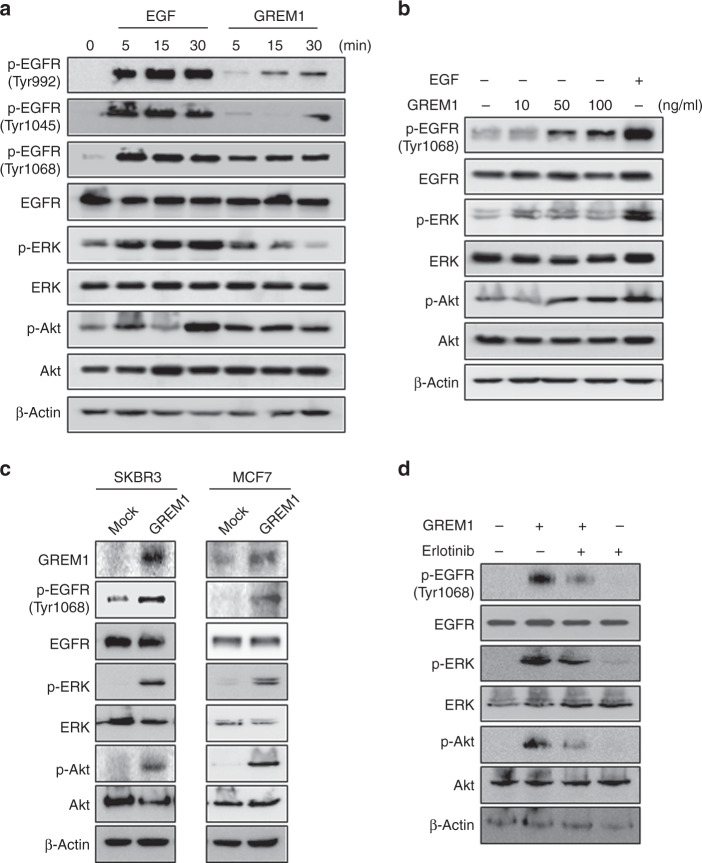

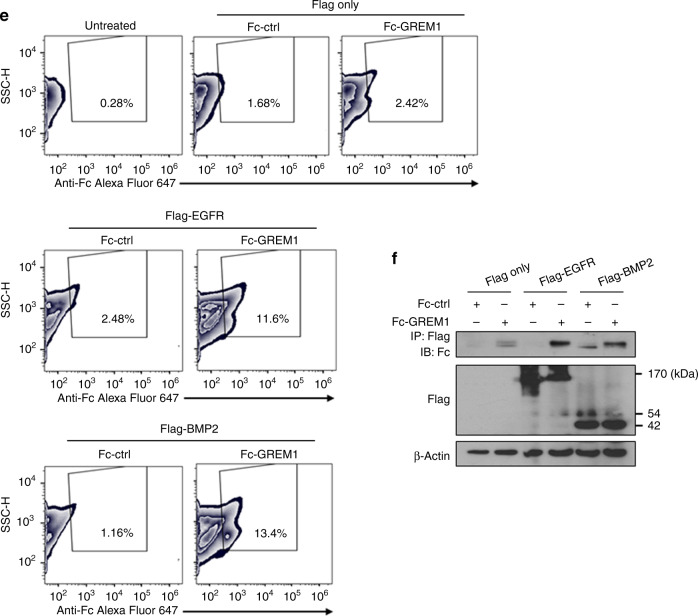
GREM1 stimulates EGFR signalling. **a**–**c** Effect of GREM1 on EGFR signalling. **a** SKBR3 cells were starved overnight and stimulated with EGF or GREM1 (50 ng/ml, each) for 5, 15 or 30 min. The lysates were then immunoblotted with the indicated antibodies. **b** SKBR3 cells were stimulated with different concentrations of recombinant GREM1 protein (10, 50 or 100 ng/ml) for 15 min. EGF (50 ng/ml) was used as a positive control, and the lysates were collected for immunoblot analysis. **c** Immunoblot assay for detection of EGFR and related signalling molecules in breast cancer cells overexpressing GREM1. Protein lysates isolated from each indicated cell were subjected to immunoblot analysis as described in ‘Methods’ section. **d** Effect of erlotinib on EGFR signalling induced by GREM1. SKBR3 cells were incubated with recombinant GREM1 protein in the absence or presence of erlotinib (1 μM) for 15 min and the lysates were immunoblotted with the indicated antibodies. **e** Flow cytometric analysis of interaction between EGFR and Fc-GREM1. HEK293 cells overexpressing Flag-only, Flag-EGFR or Flag-BMP2 were incubated with either Fc-ctrl (Fc-IgG1) or Fc-GREM1 protein for 1 h at 4 °C, followed by further incubation with Alexa Fluor 647 goat anti-human IgG (H + L) antibody for 30 min. Flow cytometric analysis was carried out using a FACS Canto II instrument. **f** HEK293 cells overexpressing Flag-only, Flag-EGFR or Flag-BMP2 were incubated with either Fc-ctrl (Fc-IgG1) or Fc-GREM1 protein for 1 h at 37 °C, and the lysates were subjected to immunoprecipitation using anti-Flag antibody. Immune complexes were immunoblotted with anti-Fc antibody.

We then determined whether GREM1 expression, regulated by ERRα, could stimulate receptors involved in breast cancer cell growth. We focused on EGFR, the upstream regulator of ERRα. Treatment of SKBR3 cells with GREM1 protein induced phosphorylation of EGFR, especially at Tyr1068, and its major target proteins, Akt and ERK, in SKBR3 cells (Fig. 5a). The GREM1-induced activation of EGFR through phosphorylation at Tyr1068 was concentration-dependent (Fig. 5b). In addition, the activation of EGFR and its downstream signalling molecules via phosphorylation was significantly enhanced in GREM1-overexpressing cells compared to that in control cells (Fig. 5c). The GREM1-induced phosphorylation of ERK and Akt as well as that of EGFR was attenuated by treatment with erlotinib, an EGFR tyrosine kinase inhibitor (Fig. 5d).

To determine whether GREM1 could bind to EGFR, HEK293 cells containing Flag-tagged EGFR were incubated with recombinant Fc-IgG1 control or Fc-GREM1 protein. As shown in Fig. 5e, Fc-GREM1, but not Fc-control protein, bound strongly to the surface of HEK293 cells overexpressing EGFR, as demonstrated by flow cytometry. Notably, the GREM1 binding to EGFR (11.6%) was as strong as the binding to the positive control, BMP2 (13.4%), and this binding was far more pronounced than that in the negative control group (Flag-only:Fc-GREM1, 2.42%) (Fig. 5e). The interaction between GREM1 and EGFR was verified by the immunoprecipitation assay (Fig. 5f). Taken together, these findings suggest that GREM1 may act as a ligand for EGFR, thereby stimulating the proliferation and growth of breast cancer cells.

### GREM1 enhances the transcriptional activity of ERRα via EGFR signalling

**Fig. 6:**
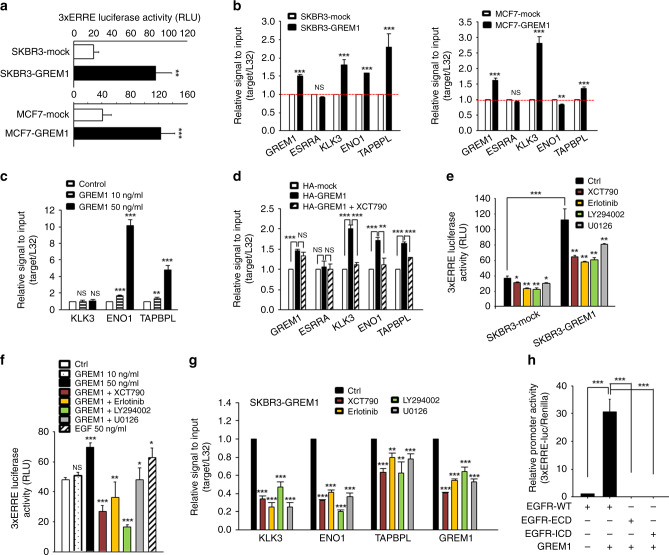
GREM1 increases the transcriptional activity of ERRα and the mRNA levels of ERRα target genes through EGFR activation. **a** Transcriptional activity of ERRα in breast cancer cells overexpressing GREM1. Each cell line was transfected with the expression vectors encoding ERRα and the coactivator PGC-1α as well as 3xERRE-luc and Renilla. After 48-h incubation, cell lysates were assayed for firefly luciferase and Renilla luciferase activities. **b** Relative mRNA levels of ERRα target genes in breast cancer cells overexpressing GREM1. The mRNA levels of the indicated genes were quantitated by qPCR analysis. **c** Effect of GREM1 treatment on the expression of ERRα target genes in MCF-7 cells. Cells were starved overnight and daily stimulated with recombinant GREM1 (10 or 50 ng/ml) for 3 days. RNA was collected and analysed by qPCR analysis. **d** Effect of GREM1 overexpression on the ERRα target gene transcription in MCF-7 cells. Cells were transfected with expression vectors of mock or GREM1 for 24 h and then incubated with vehicle or XCT790 for another 24 h. The mRNA levels of the indicated genes were measured by qPCR analysis. **e**, **f** Effect of EGFR signalling on the transcriptional activity of ERRα induced by GREM1. SKBR3-mock and SKBR3-GREM1 cells were transfected with the expression vectors encoding ERRα and the coactivator PGC-1α as well as 3xERRE-luc and Renilla for 24 h and treated with the indicated pharmacological inhibitors (XCT790, 5 μM; erlotinib, 5 μM; LY294002, 20 μM; U0126, 20 μM) for another 24 h (**e**). MCF-7 cells were transfected with the expression vectors encoding ERRα and the coactivator PGC-1α as well as 3xERRE-luc and Renilla for 24 h. The cells were pre-treated with recombinant GREM1 or EGF for 24 h and then incubated with the indicated inhibitors for another 24 h (**f**). **g** Effect of EGFR signalling on the mRNA expression of ERRα target genes in SKBR3-GREM1 cells. Cells were treated with each indicated compound for 24 h, and mRNA levels of genes were quantitated by qPCR analysis. **h** Effect of interaction between EGFR and GREM1 on the ERRα transcriptional activity. HEK293 cells were transfected with the expression vectors of EGFR (EGFR-WT, EGFR-ECD or EGFR-ICD), ERRα, PGC-1α, 3xERRE and Renilla for 48 h. The cells were incubated with recombinant GREM1 protein (50 ng/ml) for additional 1 h and analysed by firefly luciferase activity and normalised to Renilla luciferase activity. WT, wild type; ECD, extracellular domain; ICD, intracellular domain. All values in the graphs represent mean ± SD of three independent experiments. Two-sided *t* test. *P < 0.05; **P < 0.01; ***P < 0.001; NS, not significant.

After finding that GREM1 bound to and activated EGFR, we speculated that GREM1 could increase ERRα activity through activation of the EGFR signalling. In support of this postulation, the breast cancer cell lines overexpressing GREM1 had significantly higher transcriptional activity of ERRα than the respective control cell lines (Fig. 6a). Like ERRα activity, the mRNA levels of ERRα target genes, *KLK3*, *ENO1* or *TAPBPL*, were also significantly elevated in *GREM1*-overexpressing breast cancer cells (Fig. 6b and Supplementary Fig. S8a). Upon treatment with recombinant GREM1 protein in MCF-7 cells, the expression of ERRα target genes, *ENO1* and *TAPBPL*, was markedly augmented (Fig. 6c). In addition, transient transfection of MCF-7 cells with the GREM1 expression plasmid enhanced the mRNA levels of the ERRα target genes, and these effects were attenuated by the ERRα antagonist, XCT790 (Fig. 6d). The expression of *KLK3* and *TAPBPL* was also significantly inhibited in *GREM1*-knockdown cells compared to control cells, but the expression levels of *ENO1* were variable among samples (Supplementary Fig. S8b).

To further determine whether GREM1 could increase the ERRα transcriptional activity through activation of the EGFR signalling pathway, we measured the effects of the respective inhibitors of ERRα, EGFR, Akt and ERK, on the ERRα transcriptional activity increased by GREM1. First, ERRα activity was significantly elevated in SKBR3 cells overexpressing GREM1, which was suppressed by all tested inhibitors (Fig. 6e). Likewise, direct treatment with recombinant GREM1 protein increased the ERRα transcriptional activity in MCF-7 cells as much as that exerted by the positive control EGF, and this was reduced by each inhibitor (Fig. 6f). As shown in Fig. 6g, the inhibitors significantly decreased mRNA levels of the ERRα target genes in SKBR3 cells overexpressing GREM1. Further, the ligand domain of EGFR (EGFR-ECD) and the intracellular domain of EGFR (EGFR-ICD) were insufficient for GREM1 to increase the activity of ERRα and the direct interaction between GREM1 and EGFR-WT played a crucial role in ERRα activation (Fig. 6h). Overall, these results demonstrate that GREM1, as a novel target of ERRα, can potentiate ERRα activity through EGFR activation (Supplementary Fig. S9).

## Discussion

In this study, we found a novel function of GREM1 as an oncogenic protein in breast cancer growth and progression. There is evidence supporting that the aberrant overexpression of epithelial GREM1 is involved in colon cancer development and progression,^21,37^ but the role of GREM1 in pathogenesis of breast cancer remains elusive. Our present study demonstrates that GREM1 plays an important role in the growth of breast cancer cells, and the persistent upregulation of GREM1 is associated with poor prognosis in breast cancer patients. We noticed higher expression of GREM1 in ER-negative cells than in ER-positive cells. Based on this observation, we speculate that expression of GREM1 is thought to have a greater effect on cell survival or patient prognosis of ER-negative breast cancer, although expression of GREM1 may affect ER-positive breast cancer cell lines and patients. So far, very limited studies have clarified the mechanisms by which GREM1 is induced or how GREM1 affects cancer cell proliferation and growth. Tumour growth factor-β has been reported to increase the expression of GREM1,^38^ and more recently, reactive oxygen species-induced activation of nuclear factor-κB signalling has been reported to upregulate the expression of GREM1.^39^ In this study, we analysed the promoter sequences of *GREM1* and identified ERRα as an important transcription factor responsible for GREM1 expression. ERRα has been known to be essential for the growth of ER-negative breast cancer cells^33^ and hence has attracted attention as a therapeutic target in triple-negative breast cancer.^34,40,41^

GREM1 was initially known as a BMP antagonist and has been reported as a new agonist of the pro-angiogenic receptor, vascular endothelial growth factor receptor-2 (VEGFR2).^42^ GREM1 binds directly to VEGFR2 and activates the VEGFR2 signalling pathway in cultured tubular epithelial cells and renal fibroblasts.^13^ However, GREM1 has also been found to stimulate cancer cell proliferation, migration and invasion in a BMP- or VEGFR2-independent manner.^20^ GREM1 has a cystine knot structure comprising three disulfide bridges,^43^ suggesting that it is likely to act as a new ligand for distinct growth factor receptors. In the present study, we demonstrated that GREM1 physically interacted with EGFR in breast cancer cells and thereby activates EGFR signalling. Since EGFR is one of the important upstream regulators of ERRα transcriptional activity,^26,44^ GREM1 may also potentiate ERRα activity through its interaction with EGFR. Thus, as one of the target genes of ERRα, GREM1 can again activate the EGFR–ERRα axis and eventually functions as an enhancer or an amplifier for the expression of genes involved in cancer cell growth and proliferation.

As our results show, GREM1 can activate various intracellular signalling molecules such as ERK and Akt involved in cancer cell proliferation and survival. GREM1 was reported to mediate hyperplasia and the invasiveness of rheumatoid arthritis synoviocytes through activation of ERK and Akt.^45^ GREM1 activates Akt signalling to promote proliferation, migration and VEGF production in retinal pigmentation epithelial cells.^46^ In addition, GREM1 activates Smad^9^ or Slug^47,48^ to facilitate EMT and cancer cell growth. Thus, overexpression of GREM1 in cancer cells is likely to accelerate their growth and metastasis by activating distinct oncogenic pathways.

GREM1 is widely expressed in tumour-associated stromal cells and can promote cancer cell proliferation in the tumour microenvironment.^49^ Thus, GREM1 is one of the most overexpressed genes in breast tumour-associated stroma,^22^ and its level is increased in cancer-associated fibroblasts (CAFs) in basal cell carcinomas.^50^ More recently, it has been reported that CAF-derived GREM1 promotes breast cancer cell invasion.^51^ Since the importance of tumour-associated stromal cells in cancer therapy has been increasingly recognised,^52,53^ further studies are needed to precisely assess the role of GREM1 in interaction between cancer cells and tumour-associated stromal cells, such as CAFs and tumour-associated macrophages.

It was reported that antibody neutralisation of GREM1 ameliorated pulmonary hypertension in mice.^54^ MicroRNA-27b inhibits GREM1 expression by directly binding to the 3′-untranslated region of GREM1, leading to inhibition of fibrosis in pulmonary cells,^55^ and microRNA-137 negatively regulates GREM1 expression in cervical cancer cells.^56^ However, there is paucity of data demonstrating the anti-carcinogenic effects of GREM1 inhibitors. Therefore, development of chemical or biological agents to selectively targeting the aberrantly overactivated GREM1–ERRα axis merits further investigation in the context of their potential application for the treatment of breast cancer.

In conclusion, we, for the first time, report that GREM1 is a direct target gene of ERRα. Once expressed by ERRα, GREM1 activates EGFR, the ERRα upstream regulator, and ultimately augments the activity of ERRα in breast cancer cells in a positive feedback mechanism. In this context, the EGFR–ERRα–GREM1 axis can be considered to be a promising therapeutic target, especially in ER-negative breast cancer.

## Supplementary information

Supplimentary Information and Data

## Data Availability

All data and materials generated during and/or analysed during the current study are available from the corresponding author on reasonable request.
