# Supplementary material for: Gremlin-1 augments the oestrogen-related receptor α signalling through EGFR activation: implications for the progression of breast cancer
Source: Br J Cancer. 2020 Jun 23;123(6):988–99. doi: 10.1038/s41416-020-0945-0 (PMC7493948; doi:10.1038/s41416-020-0945-0)
Supplement: Supplementary file 1 — Supplimentary Information and Data [file 41416_2020_945_MOESM1_ESM.pdf]

Supplementary Methods

GREM1 enzyme-linked immunosorbent assay (ELISA)

When cells were approximately 60% confluent, they were washed with PBS and incubated in serum free medium for 48 h, followed by centrifuge for 5 min at 1,500 *g* to remove cellular materials. The collected conditioned medium was analyzed by human GREM1 ELISA kit (MyBioSource) as described by the manufacturer.

Supplementary Figure Legends

Fig. S1. GREM1 is secreted by breast cancer cells. Extracellular concentration of GREM1 in breast cancer cell lines. The conditioned medium was collected and analyzed by the human GREM1 ELISA kit.

Fig. S2. There is no significant difference in GREM1 expression between ER-negative and ER-positive patients in two Oncomine datasets. The plots of *GREM1* mRNA levels in two sets of data, TCGA breast and Ma Breast 4 were provided by Oncomine. TCGA Breast [Group 0: No value (n = 225), 1: estrogen receptor negative (n = 95), 2: estrogen receptor positive (n = 273)], Ma Breast 4 [Group 0: No value (n = 28), 1: estrogen receptor negative (n = 8), 2: estrogen receptor positive (n = 30)].

Fig. S3. The level of GREM2 is not related to the survival of patients with breast cancer. Kaplan-Meier analysis of overall survival **(a)**, relapse-free survival **(b)**, and distance metastasis-free survival **(c)** by low or high GREM2 (GREM2 probe set 220794_s_at) expression in each indicated number of breast cancer patients. OS = overall survival, RFS = relapse-free survival, DMFS = distance metastasis-free survival, ER = estrogen receptor, HR = hazard ratio.

Fig. S4. The reduced growth of shGREM1 cells is recovered by induction of GREM1 expression. **a** The cell lysates were immunoblotted with GREM1 antibody. **b** MDA-MB-453 shCtrl and MDA-MB-shGREM1 cells were transfected with mock or GREM1 plasmid (5 μg, each) for 48 h and seeded again in 96 well plate (1x 10^3/well). After 72-h incubation, the MTT assay was performed.

Fig. S5. GREM1 knockdown does not affect cell viability of normal breast epithelial cells and affects cell viability of ER-positive breast cancer cells. a The level of GREM1 expression in normal breast epithelial MCF-10A cells stably expressing shCtrl or two shGREM1 sequences (shGREM1-#1 and shGREM1-#2). Cells were selected with 1 μg/ml puromycin and the cell lyates were performed by immunoblot analysis. **b** Cells were seeded in 96-well plates and incubated for 72 h, followed by the MTT assay.

Fig. S6. The validation of lentiviral-mediated stable cell lines. a and b The cell lysates of each indicated cell line were immunoblotted with GREM1 antibody.

Fig. S7. ERRα regulates GREM1 expression. a MDA-MB-453 and SKBR3 cells were treated with XCT790 (1 or 10 μM) for 48 h and the cell lysates were immunoblotted with GREM1 antibody. d The cells were seeded in 96 well plate (1x 10^3/well) and incubated with XCT790 (1 or 10 μM) for 48 h, followed by the MTT assay. **b** MDA-MB-453 and SKBR3 cells were transfected with each indicated siRNA for 48 h, and the mRNA level of ESRRA and GREM1 were quantified by qPCR analysis. **c** SKBR3 cells were transfected with mock or ERRα in the presence of PGC1α (mock; 4 μg, ERRα; 4 μg, PGC1α; 2 μg) for 48 h and the cell lysates were immunoblotted with GREM1 antibody.

Fig. S8. GREM1 regulates the ERRα-derived gene expression. a and b Relative mRNA levels of the indicated genes measured by qPCR analysis. All values in the graphs represent mean ± SD of three independent experiments. Two-sided *t*-test. *, $P < 0.05$, **, $P < 0.01$, or ***, $P < 0.001$, NS = not significant.

Fig. S9. Model proposed to explain the role of GREM1 in breast cancer. The expression of GREM1 is increased in human breast cancer cells. Transcription factor ERRα binds directly to the promoter of *GREM1* to increase GREM1 expression. Expressed GREM1 is released and interacts with EGFR, leading to EGFR-related signaling, a major regulator of ERRα activity. The EGFR-ERRα-GREM1 axis may play a crucial role in breast cancer progression.

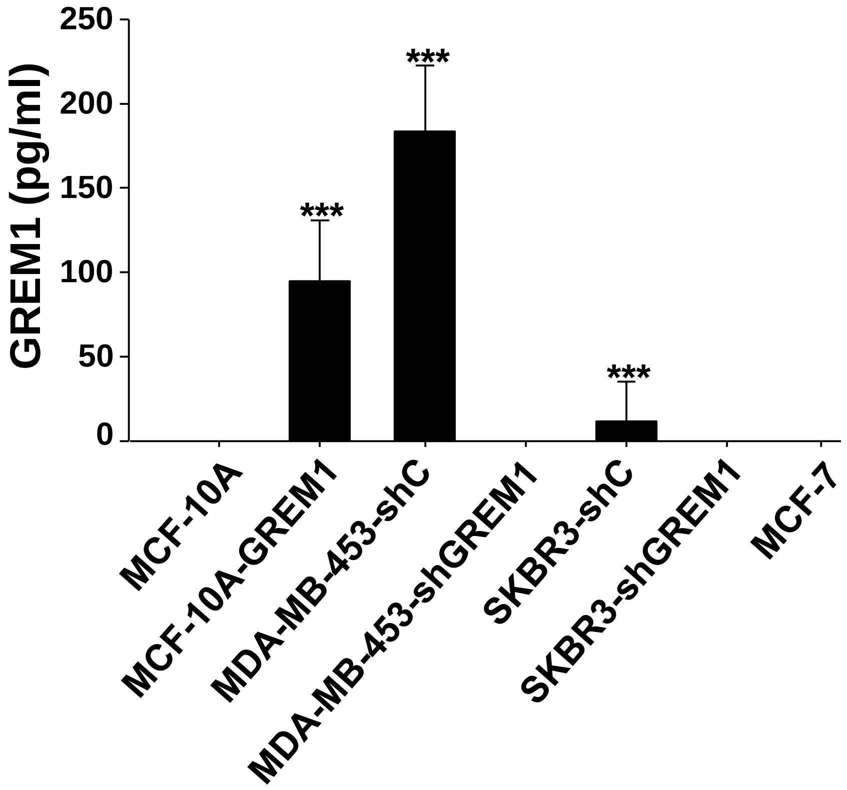

Supplementary Figure 1

TCGA Breast

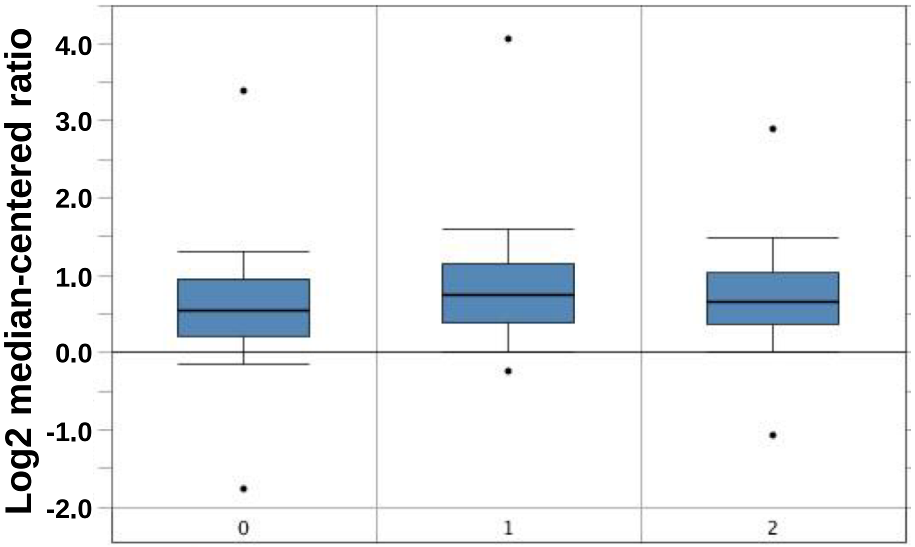

0. No value (225)

1. Estrogen receptor negative (95)

2. Estrogen receptor positive (273)

Ma Breast 4

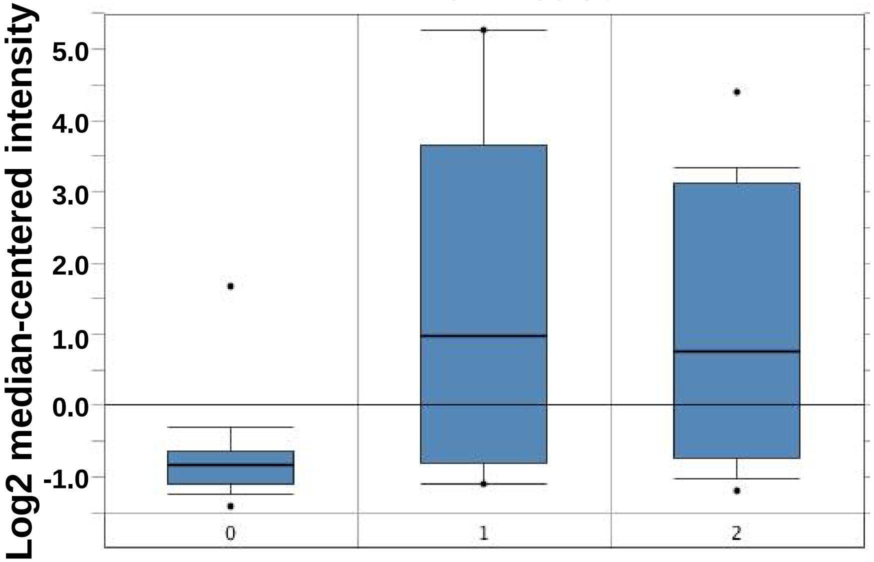

0. No value (28)

1. Estrogen receptor negative (8)

2. Estrogen receptor positive (30)

Supplementary Figure 2

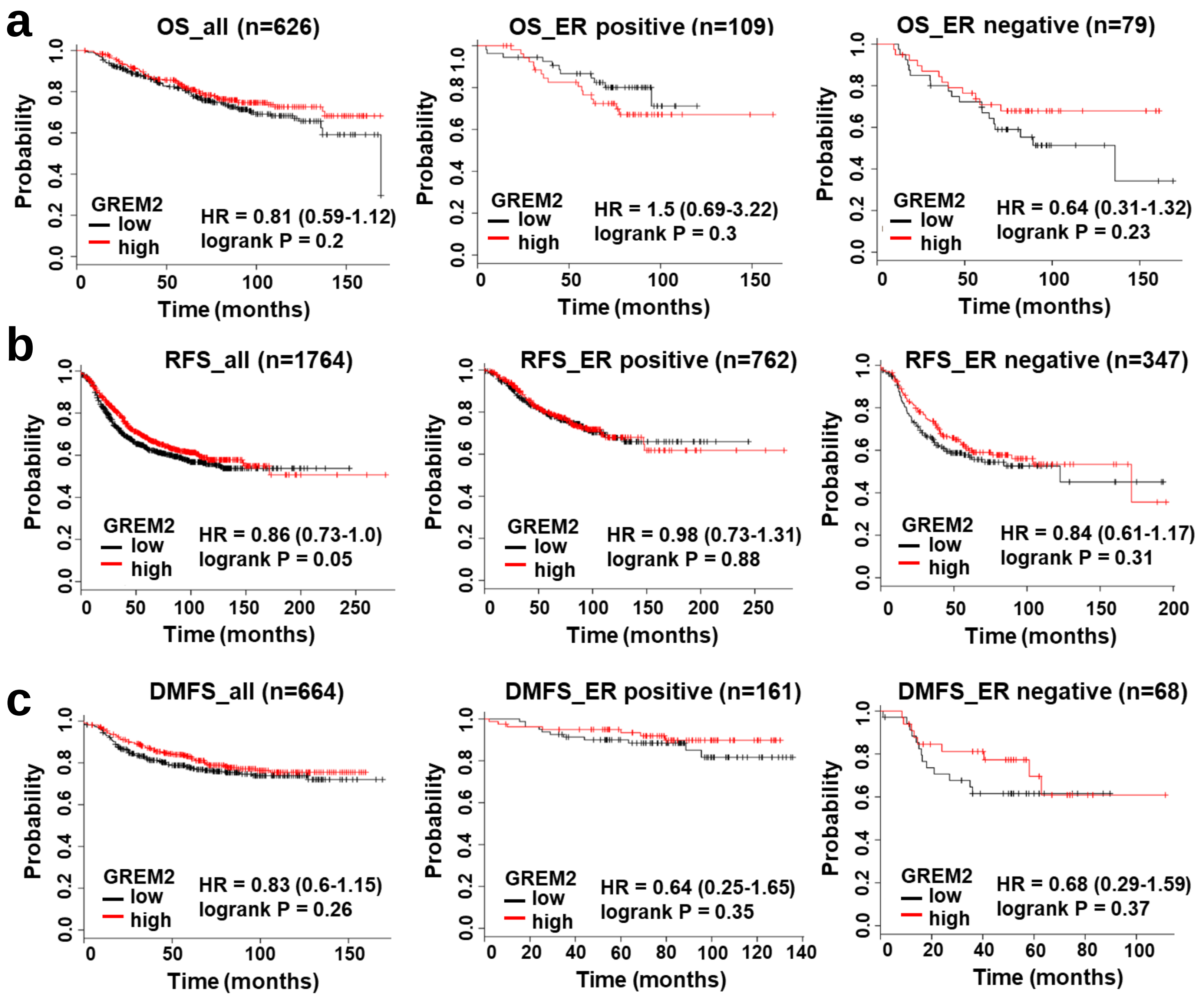

Supplementary Figure 3

a

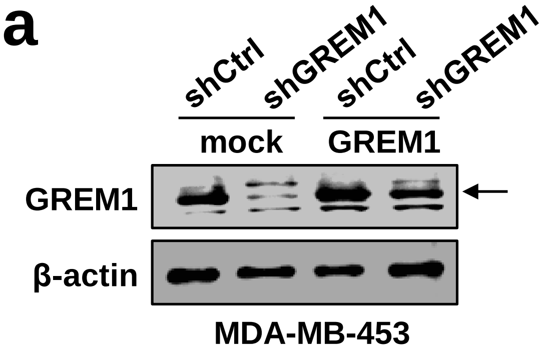

b

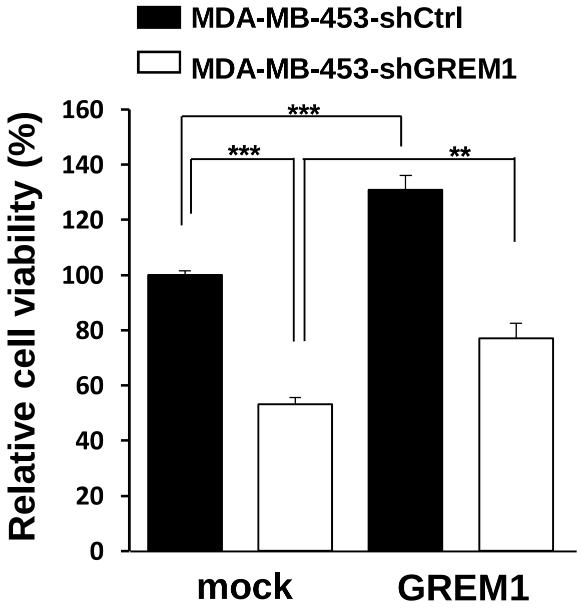

Supplementary Figure 4

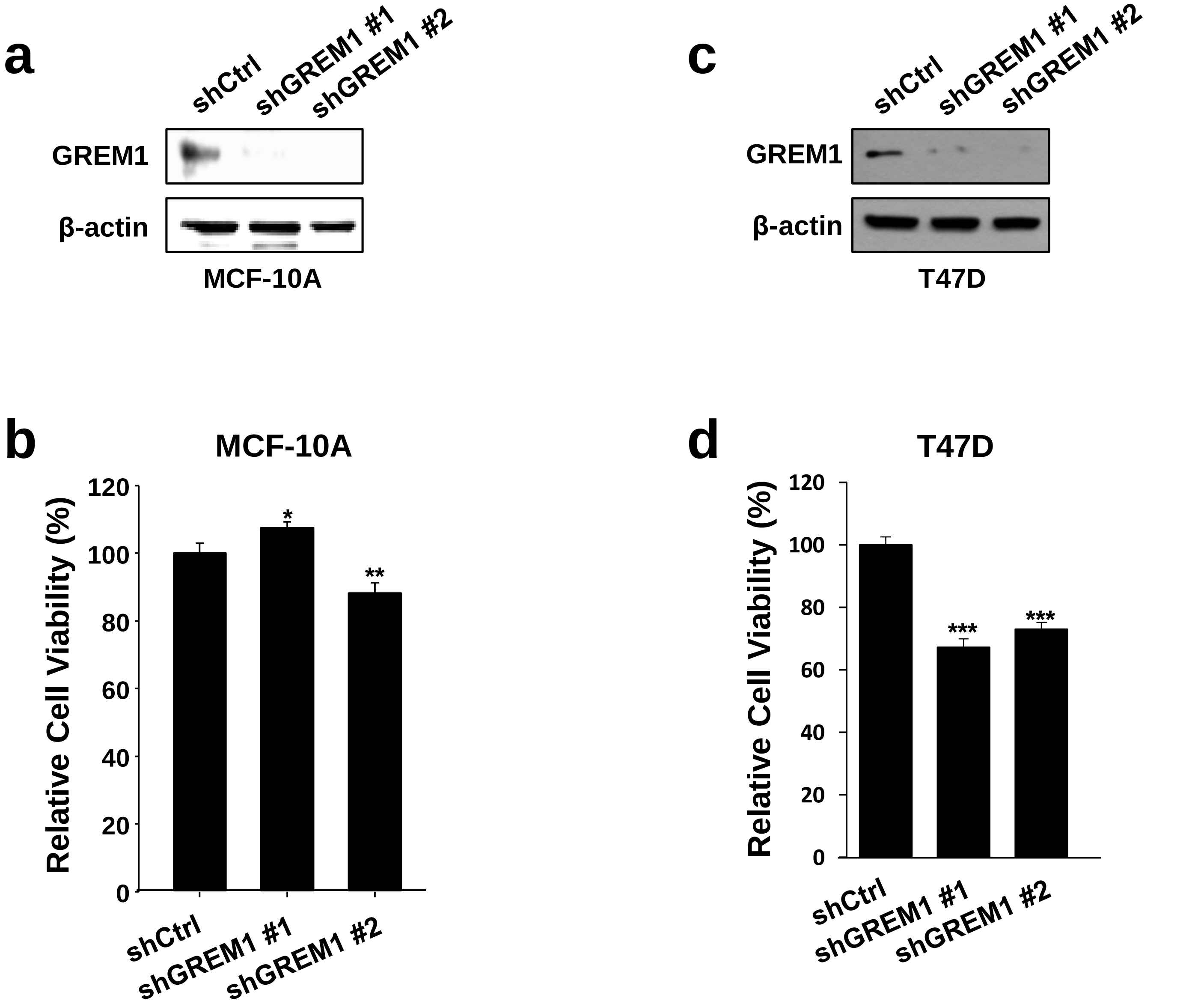

Supplementary Figure 5

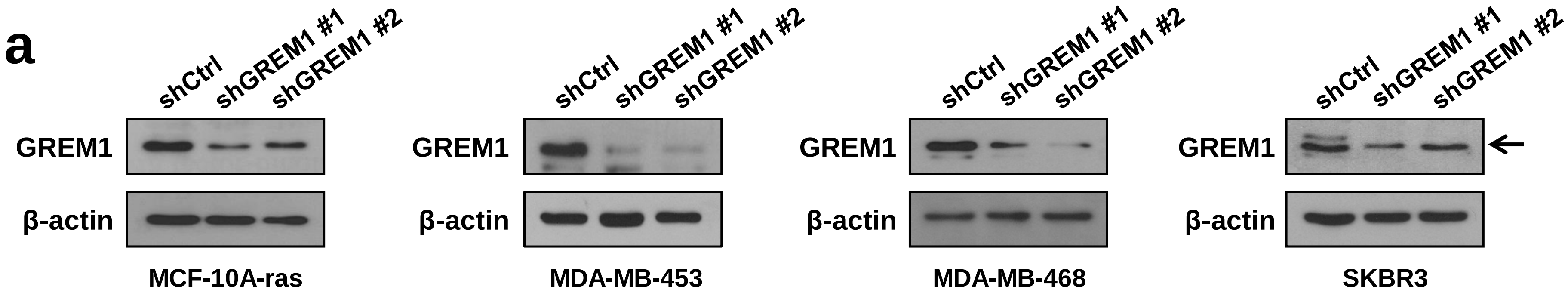

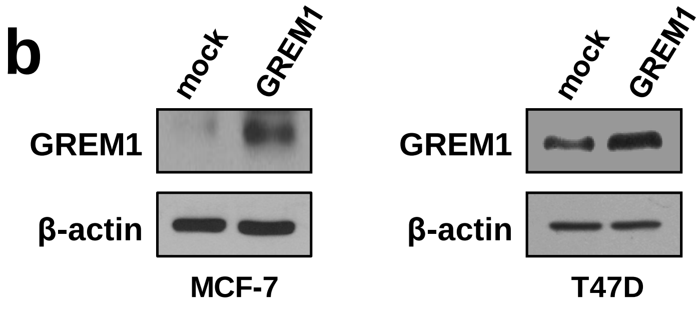

Supplementary Figure 6

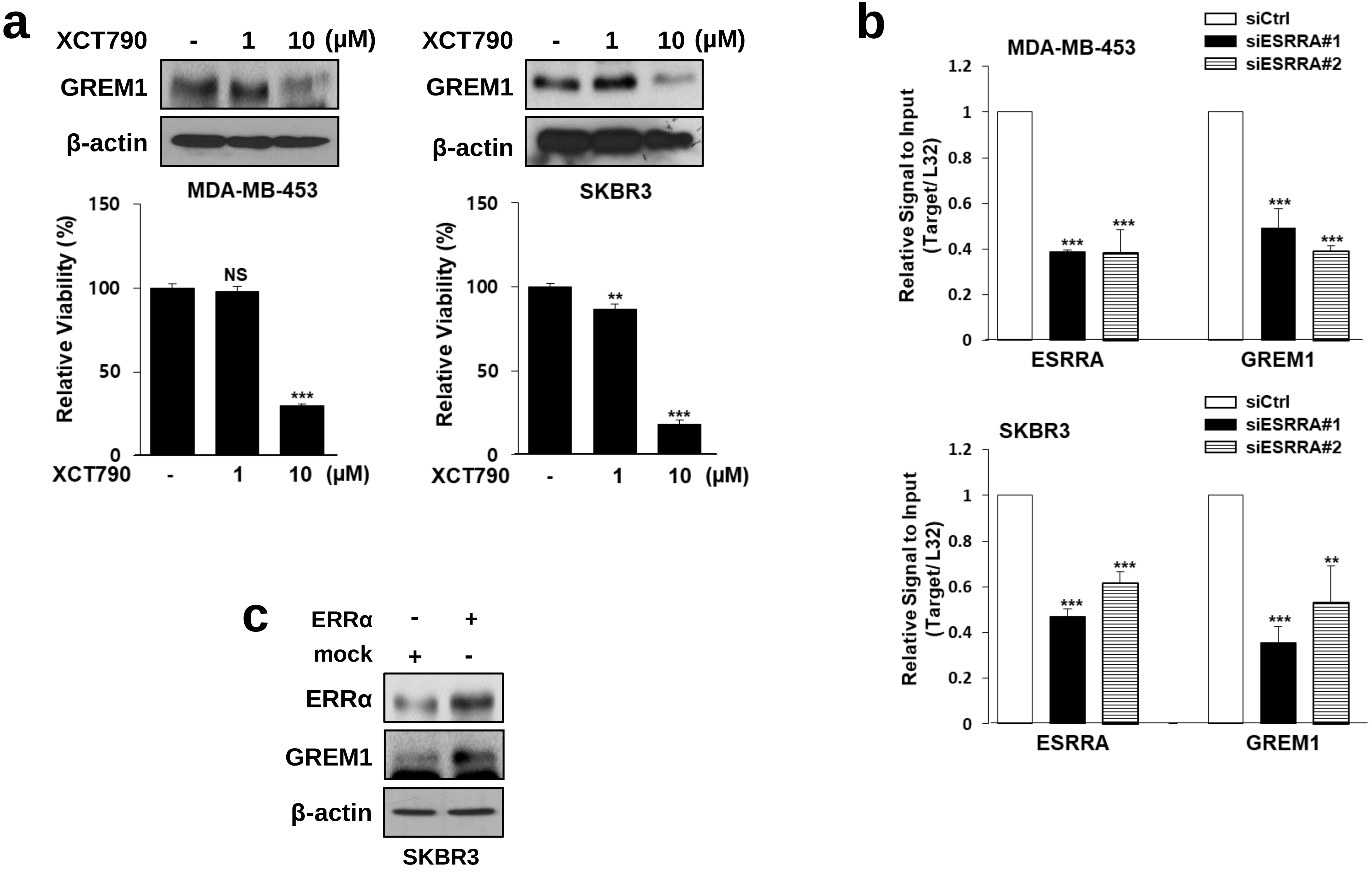

Supplementary Figure 7

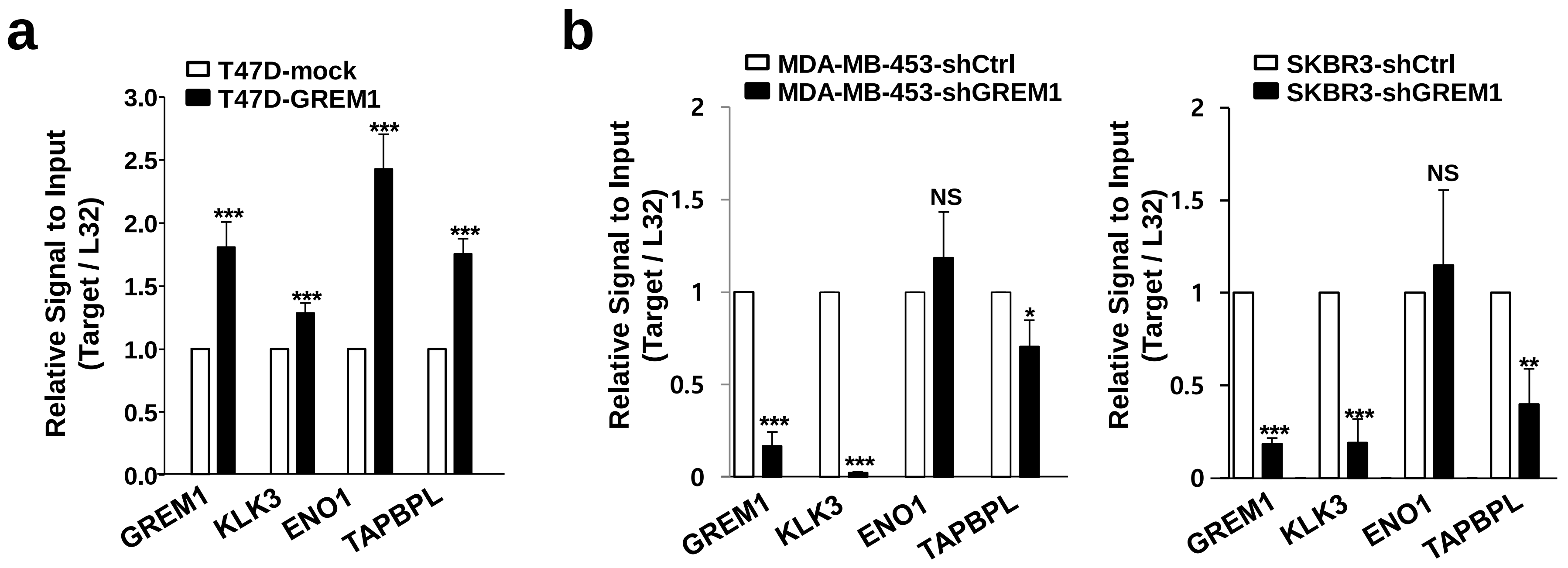

Supplementary Figure 8

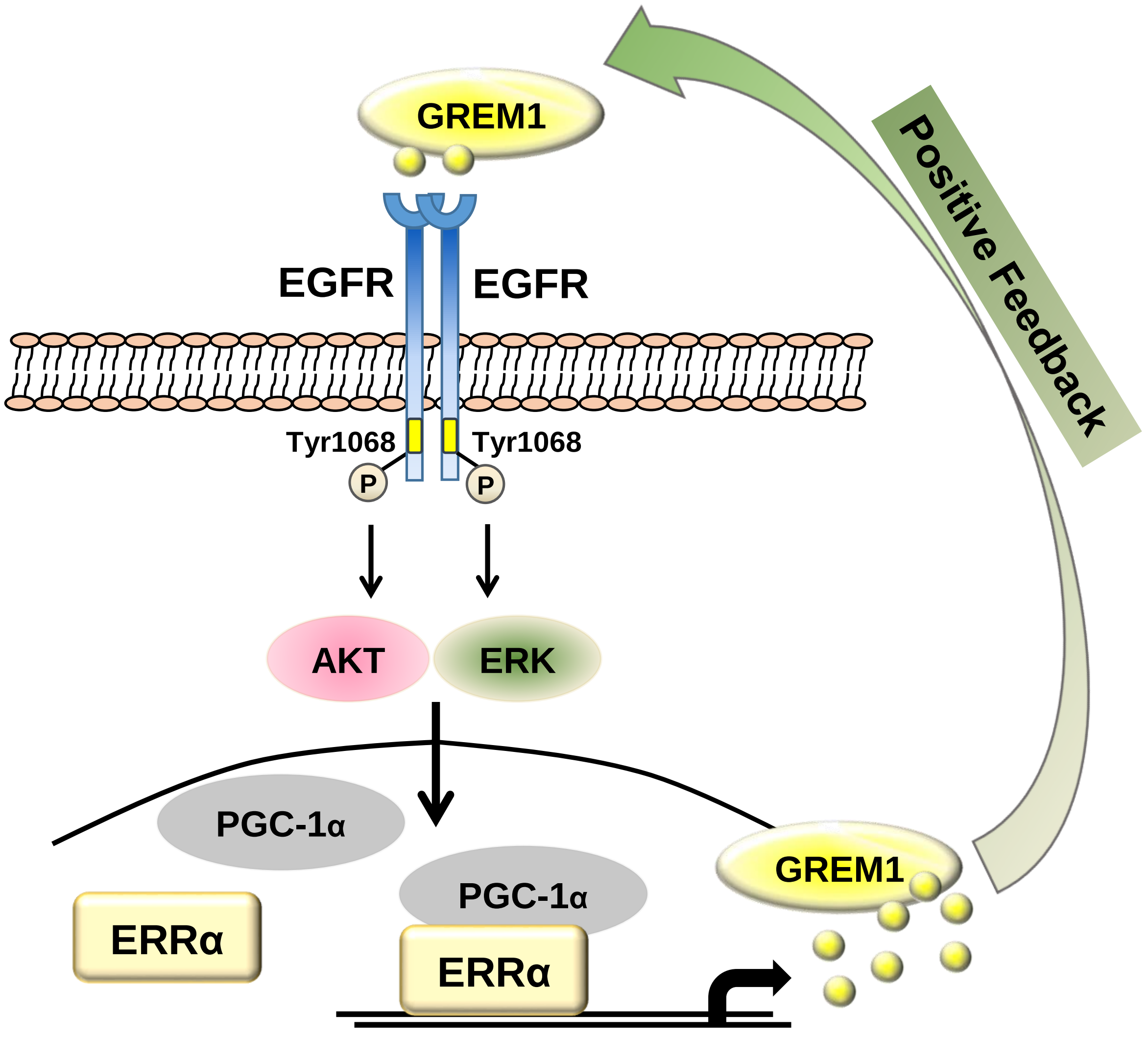

Supplementary Figure 9

Supplementary Methods

Supplementary Tables:

Table S1. qRT-PCR primer sequences

**Primers (Human)**	**Forward**	**Reverse**
GREM1	TCGAGTTGCAAGGGTTCTCT	TCTTCTCAGCCTCCTAGCCA
RPL32	CTCTTTCCACGATGGCTTTG	GTCAAGGAGCTGGAAGTGCT
ESRRA	ATCTCACACTCGTTGGAGGC	CACTATGGTGTGGCATCCTG
KLK3	ATATCGTAGAGCGGGTGTGG	TCCTCACAGCTGCCCACT
ENO1	AGTCAAAGATCTCCCTGGCA	TACGTTCACCTCGGTGTCTG
TAPBPL	CTCAGGGATTGGGTCTGTGT	GTGATGAAGACTCCCAGGGT

Table S2. ChIP assay primer sequences

**Primers**	**Forward**	**Reverse**
GREM1-‘a’	GGCATTTAAACGGGAGACG	TTGGTTTGCGGCCCTCTT
GREM1-‘b’	GATTTCCTCCCGCTCTTCC	CCTCGCCTCTTTGTCTGCT
GREM1-‘c’	GGTGTCATGTTGGCGACAG	GCCATTCTTCCCCTCTGGA
GREM1-‘d’	AACAGAAGCCTTGACTTTTGAC	AGGAGCCTGTCGCCAACAT

Table S3. Fluorescence intensity of GREM1 in tissue microarray

	-	+	++	+++	++++
	**n (%)**	**n (%)**	**n (%)**	**n (%)**	**n (%)**
**Normal breast (n=16)**	3 (18.75)	11 (68.75)	0 (0)	1 (6.25)	1 (6.25)
**ER+ breast cancer (n=17)**	0 (0)	6 (35.29)	3 (17.65)	6 (35.29)	2 (11.77)
**ER- breast cancer (n=60)**	4 (6.67)	21 (35)	14 (23.33)	11 (18.33)	10 (16.67)

Mean fluorescence intensity: < 1 (-), 1 ~ 5 (+), 5 ~ 10 (++), 10 ~ 20 (+++), > 20 (++++)

Table S4. ERRα binding site sequences

Location	Sequence
a (+62 to +84)	CCGC*TGACCCCGCGCC*GAGCCCC
b (-232 to -210)	GGGG*TGACCTCTCCCAGCCGGCC
c (-576 to -554)	AACGGGGGCGCCGG*GGTCAGCGC
d (-746 to -724)	GCCT*TGACTTT*TGACGGTCATTT

* Core sequence

Supplementary Excel File 1: List of ER-positive and ER-negative breast cancer cell lines examined for their expression of GREM1.

		Estrogen receptor status	
**#**	**Cell line**	**ER (-)**	**ER (+)**
**1**	AU565	v	
**2**	BT-20	v	
**3**	BT-474		v
**4**	BT-483		v
**5**	BT-549	v	
**6**	CAL-120	v	
**7**	CAL-148	v	
**8**	CAL-51	v	
**9**	CAL-85-1	v	
**10**	CAL51	v	
**11**	CAMA-1		v
**12**	DU4475	v	
**13**	EFM-19		v
**14**	EFM-192A		v
**15**	EVSA-T		v
**16**	HBL-100	v	
**17**	HCC1143	v	
**18**	HCC1187	v	
**19**	HCC1395	v	
**20**	HCC1419	v	
**21**	HCC1428		v
**22**	HCC1500	v	
**23**	HCC1569	v	
**24**	HCC1599	v	
**25**	HCC1806	v	
**26**	HCC1937	v	
**27**	HCC1954	v	
**28**	HCC202	v	
**29**	HCC2157	v	
**30**	HCC2218	v	
**31**	HCC2688	v	
**32**	HCC38	v	
**33**	HCC70	v	
**34**	HDQ-P1	v	
**35**	HMC-1-8	v	
**36**	Hs 281.T	v	
**37**	Hs 343.T	v	

**38**	Hs 578T	v	
**39**	Hs 606.T	v	
**40**	Hs 739.T	v	
**41**	Hs 742.T	v	
**42**	JIMT-1	v	
**43**	KPL-1		v
**44**	KPL-4	v	
**45**	MCF-7		v
**46**	MDA-MB-134-VI		v
**47**	MDA-MB-157	v	
**48**	MDA-MB-175-VII		v
**49**	MDA-MB-231	v	
**50**	MDA-MB-361		v
**51**	MDA-MB-415		v
**52**	MDA-MB-436	v	
**53**	MDA-MB-453	v	
**54**	MDA-MB-468	v	
**55**	MFM-223	v	
**56**	MX-1	v	
**57**	SK-BR-3	v	
**58**	SUM 1315M02	v	
**59**	SUM 149PT	v	
**60**	SUM 229PE	v	
**61**	SUM 52PE		v
**62**	SW 527	v	
**63**	T-47D		v
**64**	UACC-812		v
**65**	UACC-893		v
**66**	YMB-1		v
**67**	YMB-1-E		v
**68**	ZR-75-1		v
**69**	ZR-75-30		v
**Cell lines with significantly increased expression of GREM1**		22/48 (45.83%)	0/21 (0%)

Supplementary Excel File 2: Transcription factors examined for their putative binding to the GREM1 promotor

Matrix Family	Matrix	Match Total		TRANSFAC Curated Transcription Factor Targets (GREM1)	ENCODE Transcription Factor Targets (GREM1)	JASPAR Predicted Transcription Factor Targets (GREM1)	CHEA Transcription Factor Targets (GREM1)
V$CTCF	V$CTCF	9		-	O	X	X
V$BEDF	V$ZBED4	8		-	-	-	-
V$E2FF	V$E2F3	6		-	-	-	-
V$EGRF	V$EGR1	6		X	X	X	X
V$SP1F	V$SP1	6		X	X	X	-
V$PLAG	V$PLAG1	6		-	-	X	-
O$XCPE	O$XCPE1	6		-	-	-	-
V$MZF1	V$MZF1	6		X	-	-	-
V$NRF1	V$NRF1	6		X	X	X	-
V$PAX5	V$PAX5	6		X	X	X	-
V$ZF5F	V$ZF5	6		-	-	-	-
V$ESRR	V$ESRRA	5		O	X	-	-
V$KLFS	V$KLF7	5		-	-	-	-
V$MAZF	V$MAZ	5		X	O	-	-
V$HDBP	V$HDBP1	5		-	-	-	-
V$HNFP	V$HINFP	5		X	-	X	-
V$NF1F	V$NF1	5		X	-	-	-
V$E2FF	V$E2F4	4		-	X	X	X
V$E2FF	V$E2F6	4		-	O	X	-
V$AP2F	V$TCFAP2A	4	(TFAP2A)	X	X	O	O
V$MYBL	V$VMYB	4		X	X	X	X
V$CDEF	V$CDE	4		-	-	-	-
V$GCMF	V$GCM1	4		X	-	-	-
V$E2FF	V$E2F7	3		-	-	-	X
V$ZF02	V$ZBP89	3		-	-	-	-
V$ZF02	V$ZBTB7	3		X	X	-	-
V$ZF02	V$ZNF219	3		-	-	-	-
V$EGRF	V$WT1	3		O	-	-	X
V$MAZF	V$MAZR	3		-	-	-	-
V$AP2F	V$TCFAP2E	3		-	-	-	-
V$NOLF	V$EBF1	3		X	X	-	-
V$RXRF	V$VDR_RXR	3		X	-	-	X
O$TF2B	O$BRE	3		-	-	-	-
V$LTSM	V$LTSM	3		-	-	-	-
V$NKXH	V$NKX25	3		-	-	-	-
V$ZF07	V$ZNF263	3		X	x	X	X
V$E2FF	V$E2F1	2		X	X	X	X
V$KLFS	V$KLF6	2		-	-	-	-
V$KLFS	V$EKLF	2		-	-	-	-
V$KLFS	V$GKLF	2		-	-	-	-
V$ZF02	V$ZNF300	2		-	-	-	-
V$EGRF	V$EGR2	2		X	-	-	-
V$SP1F	V$SP4	2		X	X	-	-
V$PLAG	V$PLAGL1	2		-	-	-	-
V$EBOX	V$MYCMAX	2		-	-	-	-
V$EBOX	V$USF	2	(USF1)	X	O	O	-
V$ETSF	V$GABP	2		-	-	-	-
V$GLIF	V$GLIS2	2		-	-	-	-
V$GLIF	V$ZIC3	2		X	-	-	X

**V$NOLF**	V$OLF1	2		**X**	**-**	**-**	**-**
**O$MTEN**	O$DMTE	2		**-**	**-**	**-**	**-**
**O$MTEN**	O$HMTE	2		**-**	**-**	**-**	**-**
**V$HESF**	V$HELT	2		**-**	**-**	**-**	**-**
**V$HESF**	V$HES1	2		**-**	**-**	**-**	**-**
**V$HOMF**	V$HMX3	2		**-**	**-**	**-**	**-**
**V$NFKB**	V$CREL	2		**-**	**-**	**-**	**-**
**V$CP2F**	V$CP2	2		**-**	**-**	**-**	**-**
**V$NRSF**	V$NRSE	2		**-**	**-**	**-**	**-**
**V$STAT**	V$STAT3	2		**X**	**X**	**X**	**x**
